# Distal Loop Flexibility of a Regulatory Domain Modulates Dynamics and Activity of C-Terminal Src Kinase (Csk)

**DOI:** 10.1371/journal.pcbi.1003188

**Published:** 2013-09-05

**Authors:** Sulyman Barkho, Levi C. T. Pierce, Maria L. McGlone, Sheng Li, Virgil L. Woods, Ross C. Walker, Joseph A. Adams, Patricia A. Jennings

**Affiliations:** 1 Department of Chemistry and Biochemistry, University of California at San Diego, La Jolla, California, United States of America; 2 Department of Medicine, University of California at San Diego, La Jolla, California, United States of America; 3 San Diego Supercomputer Center, University of California at San Diego, La Jolla, California, United States of America; 4 Department of Pharmacology, University of California at San Diego, La Jolla, California, United States of America; Stanford University, United States of America

## Abstract

The Src family of tyrosine kinases (SFKs) regulate numerous aspects of cell growth and differentiation and are under the principal control of the C-terminal Src Kinase (Csk). Csk and SFKs share a modular design with the kinase domain downstream of the N-terminal SH2 and SH3 domains that regulate catalytic function and membrane localization. While the function of interfacial segments in these multidomain kinases are well-investigated, little is known about how surface sites and long-range, allosteric coupling control protein dynamics and catalytic function. The SH2 domain of Csk is an essential component for the down-regulation of all SFKs. A unique feature of the SH2 domain of Csk is the tight turn in place of the canonical CD loop in a surface site far removed from kinase domain interactions. In this study, we used a combination of experimental and computational methods to probe the importance of this difference by constructing a Csk variant with a longer SH2 CD loop to mimic the flexibility found in homologous kinase SH2 domains. Our results indicate that while the fold and function of the isolated domain and the full-length kinase are not affected by loop elongation, native protein dynamics that are essential for efficient catalysis are perturbed. We also identify key motifs and routes through which the distal SH2 site might influence catalysis at the active site. This study underscores the sensitivity of intramolecular signaling and catalysis to native protein dynamics that arise from modest changes in allosteric regions while providing a potential strategy to alter intrinsic activity and signaling modulation.

## Author Summary

The Src family of protein kinases (SFKs) are integral in many cellular signaling pathways. Aberrant SFK activity correlates with the development of various cancers and autoimmune diseases. Csk regulates these kinases via phosphorylation of a tyrosine residue on the C-terminal tail of SFKs, leading to suppression of activity. Csk's SH2 domain is essential for function and understanding how sites within this domain influences Csk activity is of paramount importance. A structural divergence can be noted in the unique presence of a tight turn in Csk's SH2 domain when compared to the longer loops found in homologous kinase domains. We sought to study the importance of flexibility in this surface exposed, distal loop to the kinase's functional motions and found that enzyme activity and dynamics are sensitive to modifications at this site. Our complementary use of statistical computational methods coupled with structural and functional experimental methods helped reveal routes of intramolecular and long-range communications along Csk's framework. These insights may provide a general framework for identifying potential sites for specific targeting by design that aid in kinase activity modulation – a therapeutic strategy of great importance and interest.

## Introduction

Many cell functions are under the regulatory control of the Src family of tyrosine kinases (SFKs). These multidomain kinases are typically localized to the plasma membrane in their active forms where they phosphorylate numerous protein substrates associated with cell growth, differentiation, adhesion, motility and invasion [1]–[4]. In healthy cells, SFKs are under tight regulatory control and are only transiently activated upon stimulation; however, Src, the prototype for the SFKs, is often overexpressed or unregulated in many cancers and accordingly is targeted for drug chemotherapy [5]–[7]. The modular Src Homology (SH) SH2 and SH3 domains in SFKs assist in keeping such tight intramolecular control over kinase activity [8].

In addition to the auto regulatory function of the SH domains, the C-terminal Src Kinase (Csk) serves as the master regulator and suppressor of SFKs and thus, plays an essential role in terminating SFKs' functions. Csk phosphorylates a single tyrosine (Tyr-527) in the C-terminal tail of c-Src, the cellular form of Src. This phosphorylation event induces extensive changes in c-Src that result in domain rearrangement and the generation of a low activity form [9]–[10]. Further down-regulation of SFKs is assisted by dephosphorylation of a single tyrosine (Tyr-416 in c-Src, Tyr-394 in Lck) in the activation loop of the kinase domain of the SFKs [11]–[12]. While several protein phosphatases have been implicated in this role, a recent study suggests that LYP interacts directly with Csk and upon dissociation is competent to dephosphorylate Tyr-394 in the SFK Lck in T cells [13].

**Figure 1:**
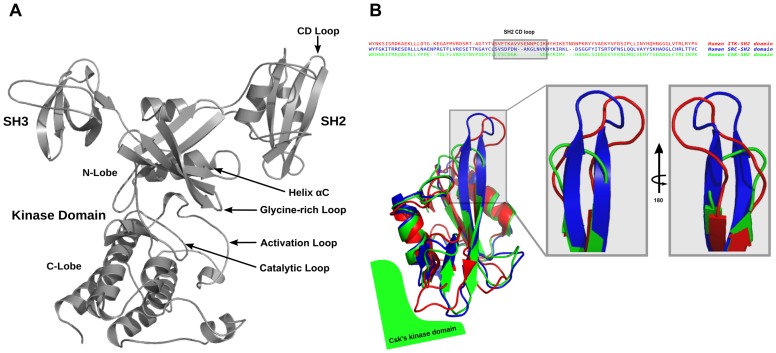
Sequence and structural alignment of homologous SH2 domains. (A) Structure and domain arrangement in full-length Csk (PDB ID 1K9A). (B) Sequence and structural alignment of SH2 domains of homologous protein kinases: Itk (red), Src (blue), Csk (green) illustrating the stark differential loop lengths among these domains. Most notably, Csk possesses the shortest CD loop (turn) of the three modular domains. Sequence alignment was performed using Clustal Omega through UniProt web server. Structural alignment was performed in PyMol using PDB entries 2ETZ, 2SRC, and 3EAC for Itk, Src, and Csk, respectively.

Like its substrates, Csk also possesses N-terminal SH2 and SH3 domains (Figure 1A) [14]. A notable difference between SFKs and Csk is the tertiary arrangement of the SH domains [15] and the absence of activation loop phosphorylation in Csk. The kinase domain of Csk requires activation by the peripheral motifs and SH2 and SH3 domains, whereas these domains are important for localization and activity repression in SFKs via binding the phosphorylated tail [15]. Biochemical and structural characterization of SH2 and SH3 modules of many proteins is abundant in the literature as these domains have been utilized extensively to investigate protein folding and stability [16]–[17], thus providing a rich database that enables further nuanced exploration. Recently, discoveries of unique and non-canonical modes of intramolecular dynamic regulation by these domains [18] may be taken as evidence that our understanding of how modular kinase domains collectively function within a macromolecular assembly remains in its infancy.

As Csk orchestrates the activity of all SFKs, the quest to understand how the non-catalytic domains in Csk enhance its catalytic efficiency is of paramount importance. The classical approach taken in previous studies is based on modifying predicted or known interdomain contacts between the SH modules on one hand and the kinase domain on the other [19]–[23]. These studies successfully identified several key residues and motifs essential for relaying signals from peripheral regions to the active site. The SH2 domain is central to Csk's function whether it is direct activation via the small kinase lobe interactions on the interface [21], [23] or the binding of phosphotyrosine ligands like Csk Binding Protein (PAG/CBP) that was shown to transmit its activating signal via the SH2-Kinase linker [24]. In addition, mammalian Pragmin and bacterial peptide effectors possessing the EPIYA sequence competitively interact with Csk via the SH2 domain and binding of the bacterial motifs may lead to Csk's recruitment to the membrane and down-regulating SFKs to facilitate infections [25]. One important allosteric site within the SH2 domain is its unique disulfide bridge whose redox state can provide further intramolecular activity modulation [26]. Perturbations to these sites within the SH2 domain or its flanking linker regions, which are removed from the active site, produce effects in local and global flexibility and in turn influence enzymatic efficiency. A close inspection of homologous kinases shows subtle yet notable differences between the SH2 domains of tyrosine kinases. Most significant examples are shown for Csk, Src and Itk in Figure 1B. While the global fold and structural elements in the domain are conserved, distinct differences are apparent in loops/turns connecting the central antiparallel beta strands. Given the fact that All-atom MD suggested communication between the CD/DE loop and that tethering the Csk SH2 domain is important in regulating activity [26], our current approach involved emulating a more flexible CD loop as observed in Src and Itk in the SH2 domain of Csk. For the latter kinase, proline-mediated switching of the loop conformation was shown to be responsible for conformer-specific ligand recognition [27]. Importantly, our approach does not interrupt native contacts between the SH2 and kinase domain and preserves the overall fold and domain arrangement of Csk. Using glycine insertion has the added advantage of introducing loop flexibility while minimizing the impact on secondary structure formation.

Using a combination of theoretical and experimental techniques on the isolated domains and the intact protein, we found that our loop variant did not alter the global fold of the isolated SH2 domain or full length Csk. However, the double glycine insertion in the SH2 domain loop at a site greater than 45 Å from the active site reduces the catalytic activity. Given that PAG/CBP's interaction with wild type Csk via the SH2 domain does not affect the catalytic rate but the apparent affinity of Csk towards Src is increased [24], the SH2 domain can potentially serve both as a hub for *activating* and *deactivating* the kinase. Taken together, we show experimentally that a seemingly subtle distal perturbation to the framework of the enzyme can lead to reduction in catalytic activity while our computational methods outline the network paths that may facilitate such long-range communication between remote regions in the enzyme.

## Results/Discussion

### Stability and Folding of the SH2 Domain: Loop Elongation Preserves Global Fold of Isolated SH2 Domain but Affects Its Dynamics and Stability

The variant SH2 domain was generated by inserting two glycines between residues Gly124 and Lys125 using site-directed mutagenesis protocols. Chemical denaturant-induced equilibrium unfolding studies on the expressed variant indicate that elongating the CD loop in Csk's SH2 domain does not interfere with the ability of the isolated domain to fold. A comparison plot of the fraction of folded protein (F_app_) as a function of final denaturant concentration for the wild type and variant SH2 domains is given in Figure S1. A cooperative unfolding transition is observed for both the wild type and the variant SH2 domains. The variant shows a slight destabilization with respect to the wild type domain, with transition midpoints of 2.9M and 3.6M Guanidine-HCl, respectively, and a calculated destabilization free energy of ∼1.5 kcal/mol (S1). This small apparent change in stability does not suggest a change in the folding of the domain. To verify that the global fold of the SH2 domain remains intact, we employed ^1^H-^15^N Heteronuclear Single Quantum Correlation (HSQC) NMR spectroscopy. An overlay view of HSQC spectra of the two domains is given in Figure S2. As can be seen in this plot, the overall chemical shift pattern observed for the SH2 domain variant is similar to that observed for the wild type domain and is indicative of a well-folded protein. The largest chemical shift changes observed in the variant map to the site of insertion and nearby motifs. The integrity of the unique disulfide bridge between C122 and C164 remained intact as judged by chemical modification and by the unique cysteine beta carbon chemical shifts [28].

While the SH2 domain structural fold was maintained upon insertion, this modification alters the global stability of the intact domain. The structural determinants of this destabilization were further explored by monitoring site-specific Hydrogen-Deuterium exchange (HDx) rates for individual amide protons within the two domains by NMR. This experiment involves initiating an HDx time course by placing the purified domain in ∼99% deuterated buffer and monitoring the change in intensities of backbone amide resonances over time by recording a series of sequential ^1^H-^15^N HSQC spectra. Plots of resonance intensity over time are indicative of the stability of individual sites (see [29] and references therein for full review) and can be compared between wild type and the variant domain. The results of these studies are shown in Figure S3. Areas colored in red indicate destabilization, blue indicates stabilization, and yellow is for no change in protection against exchange. Changes in exchange rates are observed not only around the CD loop region (insertion site) as expected but also distal to this site with deprotection observed for probes that map to the α-helix and SH2-Kinase interface (surrounding the DE loop) whose hydrophobic minicore is known to be important for cross-domain communications [21], [23] and is a good candidate for potential allosteric regulation of kinase activity. In general, global domain destabilization observed in folding studies (Figure S1) is not manifested by global faster exchange as might be expected but revealed as a more complex mix of faster exchange together with unaffected as well as stabilized sites within the central beta sheet and interacting α-helices. This behavior was also observed previously in another context, and may be a reflection of the unique role of Csk's SH2 domain in relaying information from the periphery to the central active site within the intact kinase [26], [30]. In an effort to determine whether incorporation of the Gly-Gly insertion into the SH2 domain in the *full length protein* altered the conformation of the kinase, circular dichroism (where the signal is dominated by the large lobe of the kinase domain) and further native state HDx studies (see below) were conducted. The data indicate the full length kinase's helical content is maintained by the double glycine insertion as both the wild type and variant Csk proteins show equal molar ellipticities with overlapping double minima (Figure S4).

### Steering the Kinase Activity by Distal Loop Elongation

Proximal regions to the active site have significant influence on the catalytic activity of protein tyrosine kinases [31]. For example, the activation loop in Csk, which lacks a phosphorylation site, maintains functional importance as an inhibitory structure within Csk's catalytic machinery [32]–[33]. In addition, an interesting observation from the crystal structure [34] of Csk is the presence of two distinct conformations for the SH2 domain. The “down” conformation indicates extensive SH2 domain contacts with the small lobe of the kinase domain and was proposed to influence the ordering of the functionally important αC motif. Further exploration of proximal regions via site-directed mutagenesis and alanine scanning studies has identified contacts that are deemed essential for kinase activation via the SH2 domain [21], [23]. As a modular enzyme, Csk's domain linkers also play key roles in domain packing and arrangement of the active kinase conformation [15]. A specific site in the SH2-kinase linker (residue 183), which packs against the small lobe of the kinase domain, is very sensitive to the size of its side chain (Phe in wild type Csk) and is essential for functional flexibility and Csk activation via the SH2 domain [24]. These studies formed the framework for understanding how docking interactions between regulatory sites coordinate to control catalysis in Csk.

**Figure 2:**
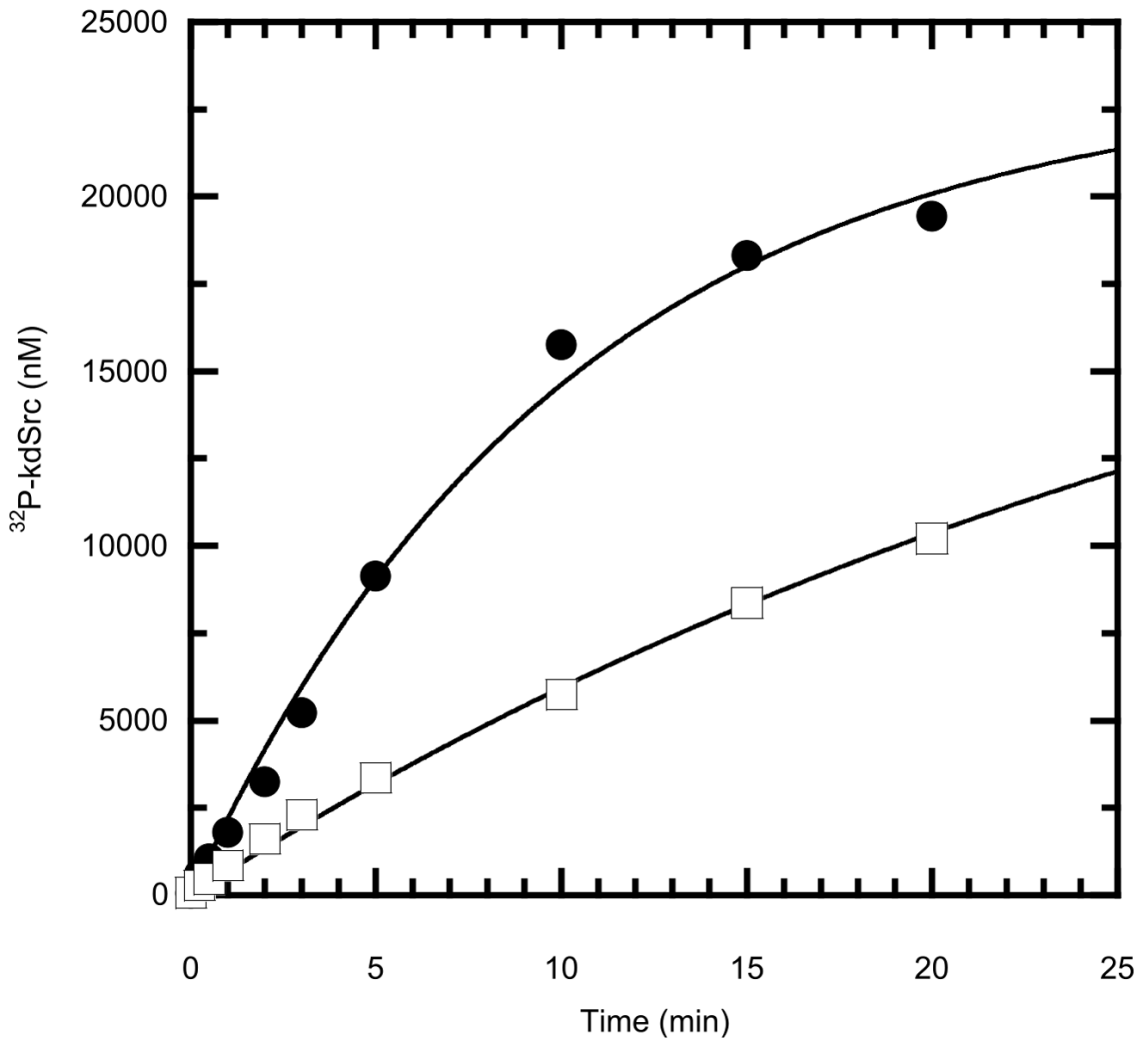
CD loop elongation in the SH2 domain results in reduced kinase activity of full length Csk towards its physiological substrate Src. Wild type Csk's (circle) and the variant's (square) kinase activity was monitored in a [γ-^32^P]ATP coupled radioactive assay in which a kinase dead substrate (kdSrc) is phosphorylated as a function of time. The reactions typically included 200 nM Csk, 20 µM kdSrc, and 50 µM ATP at 23°C.

In a departure from the investigation of directly coupled functional motifs in regulating activity, we identified that the redox state of the unique disulfide bridge in the SH2 domain of Csk (Figure S3) that links the CD loop to the SH2 kinase linker modulates kinase activity [26]. This important allosteric site is 45 Å removed from the active site. In the current study, we focused on the properties of solely the CD loop region in the SH2 domain as this motif shows significant variance among homologous kinases (Figure 1B). Interestingly, in Itk this loop is an important motif for recognition gating [27]. Rather than adaptor recognition, we hypothesized that this loop would be an important regulator of the active site in Csk based on earlier theoretical and experimental studies [26]. To determine the effects of loop elongation on substrate and scaffold binding as well as kinase activity we employed a variety of kinetic and thermodynamic techniques. Csk's time-dependent phosphorylation of its physiological substrate, Src, was monitored in a [γ-^32^P]-ATP radioactive assay. In all assays, a kinase defective Src (kdSrc) is used to eliminate the possibility of substrate autophosphorylation and interference [35]. A plot of the γ-^32^P incorporation into Src as a function of time for both Csk proteins is given in Figure 2. Incorporation of γ-^32^P into kdSrc for the variant was reduced by approximately 3-fold compared to wild type Csk. Consistent with the observation that Csk utilizes substrate-assisted catalysis [36], phosphorylation of the generic poly(Glu_4_Tyr) kinase substrate is also reduced, though to a lesser extent (Figure S5).

The slower observed rate of phosphorylation by the variant Csk could be a function of reduced substrate binding and/or catalysis. To determine what limits catalysis in the variant, we performed comparative studies for substrate binding. Plots of the observed rate as a function of kdSrc and ATP binding for the wild type and variant are given in Figure S6. The affinities for either phosphoryl accepting or donating substrates are unaffected (within error) upon insertion. Therefore, enzyme-substrate complex formation is not responsible for the observed rate reduction in the variant. In addition, a peptide derived from the membrane-anchored Csk binding protein (CBP), which up regulates Csk by binding to its SH2 domain, activates both the wild type and variant enzymes to the same extent (Figure S7) and the measured phosphopeptide dissociation constants for both enzymes are within error. Thus, the loop elongation does not appear to have a significant effect on how CBP activates Csk via the phosphotyrosine-SH2 interaction. Since maximum turnover is limited by a conformational change in Csk [36], we suspect that the decreased rate for the variant is the result of changes in overall protein dynamics.

### Deuterium Exchange Mass Spectrometry (DXMS) of Native State Dynamics

Previous studies have shown that reduced flexibility in Csk can result in dramatic decreases in kinase activity (Phe183Gly). In addition, alterations in stability can perturb the interconversion between active and inactive forms of Csk within the native ensemble [37]. To determine if the overall conformational ensemble is altered in Csk we utilized DXMS methods to explore the native state dynamics of the enzyme and its variant. In this experiment a fragmentation map is established for the protein as described [38]. The non-deuterated protein is exposed to deuterated buffer for varying amounts of time, and the mass of each peptide-probe is measured as a function of incubation time to determine the number of in-exchanged deuterons incorporated with respect to a fully protonated and deuterated probe (see Materials and Methods). Csk and the variant were first treated with a protease for digestion and the resulting peptide fragments were identified by sequence matching ([39], Materials and Methods); both proteins produced nearly identical fragmentation patterns, which allowed for optimal analysis by DXMS. Incubation of the proteins in deuterated buffer led to increases in mass envelopes of identifiable peptides due to deuterium incorporation as shown for a representative probe from the variant and the wild type protein in Figure S8. These time-dependent analyses were performed on fragments that cover most of the protein's sequence.

**Figure 3:**
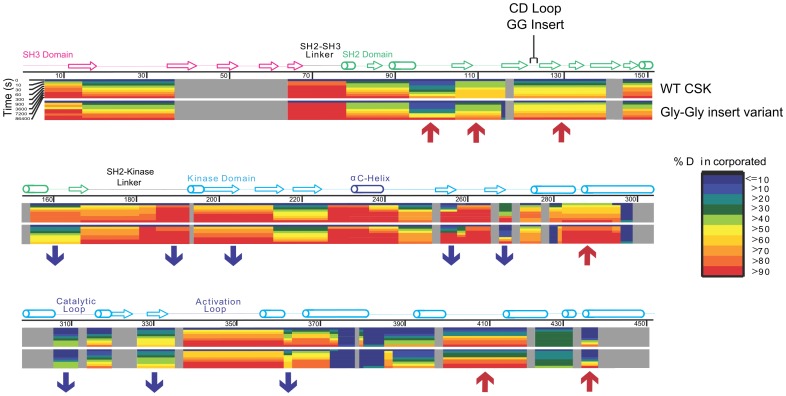
Heatmap schematic of % Hydrogen-Deuterium exchange showing relative amide proton protection from D_2_O solvent in full length variant and wild type Csk. Color-coded blocks show levels of deuteration for peptide probes in wild type Csk (top row) and the variant (bottom row) as indicated on the right. Regions of secondary structure motifs and domains identified from the crystal structure are shown above the sequence. The figure was generated by mapping all reliably-identified peptides onto Csk's wild type sequence then overlaying the resulting blocks with exactly matching peptides that have the same mass, charge and retention times in LC-MS analysis for both proteins. The level of protection is indicated by coloring each peptide at any given time-point based on the maximum percentage of deuterons incorporated according to the key on the right. Observed differences are indicated with red upward arrows for segments that show relative deprotection while blue downward arrows indicate relative protection in the variant with respect to wild type. A difference greater or equal to 10% in protection is considered significant if observed in at least two time points. Gray blocks indicate absence of reliable probes for that part of the sequence. Peptide identification and analysis were performed after combining two independent pools of generated peptides for each of the wild type and variant Csk for verification.

**Figure 4:**
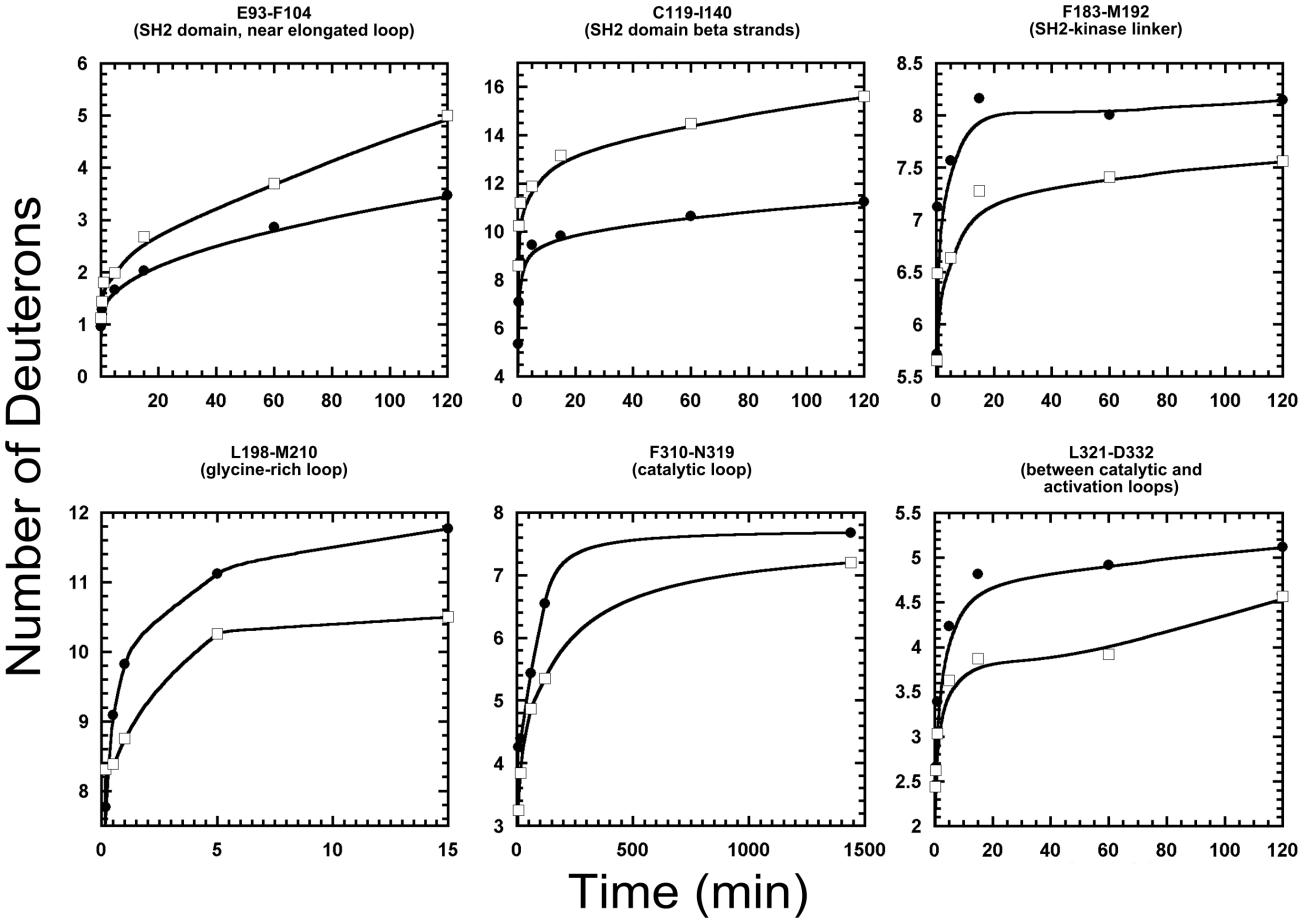
Effects of CD loop elongation on Time-dependent solvent Deuterium incorporation into Csk peptide probes. Deuterium incorporation into several probes in wild type (circle) and variant (square) Csk is plotted as a function of time. Data were obtained over time courses of 1440 minutes in deuterated buffer. Only relevant time frames are displayed for comparison and clarity. In general, a probe mass difference of 1 deuteron is considered significant between wild type and the variant if observed in at least two time points. Peptide identification and analysis were performed after combining two independent pools of generated peptides for each of the wild type and variant Csk for verification. Residue name and numbering are based on the wild type sequence.

Comparative color-coded heat maps [40] for the variant and wild type are given in Figure 3 and illustrate the relative deuteration levels for the detected probes in both wild type and variant Csk. Several significant regions of the protein are influenced by the insertion and clearly show differences in deuterium incorporation. The blue and red arrows in Figure 3 indicate some of these notable segments. An expected difference is noted for the regions flanking the insertion site in the CD loop and show faster exchange relative to the wild type protein. The sequence in this region corresponds to the C, D, and E beta strands of the SH2 domain, which agrees with the residue-specific HDx profiles from the NMR-detected exchange on the isolated domains. Slower exchange (consistent with stabilization) is noted for peptides that are part of the kinase domain of Csk. Plots of the number of deuterons as a function of time for specific motifs that are important for localization, regulation and kinase activity are given in Figure 4. Regions in which dynamics are affected by the CD loop elongation include *deprotection* in the flanking strands around the insertion site (βC and βD) and in the alpha helix of the SH2 domain in contact with kinase linker. *Protection* is observed in the SH2-kinase linker, the beta structure of the nucleotide-binding lobe of the kinase domain, specifically, the phosphate binding motif (glycine-rich loop), the hinge linking the two kinase lobes, and the activation loop all show slower solvent deuterium incorporation in the variant. The absence of the activation loop from the crystal structure is indicative of its high mobility in the wild type protein and this is also observed in our DXMS studies as probes from the loop are readily deuterated to >50% within the first deuteration time point (10 s) and computational studies agree with this interpretation (see below). Although experimental data we provide here indicate that Csk's catalytic efficiency in kdSrc phosphorylation is likely reduced due to disruption of coordinated motions in the native ensemble that are part of the catalytic machinery, the routes for such long range effects need to be elucidated. Therefore, we turned to recent advances in computational methodology and analysis [41] with additional tools to address that question with atomic details.

### Experimental and Computational Studies Reveal Trans-Domain Crosstalk Important for Catalysis

**Figure 5:**
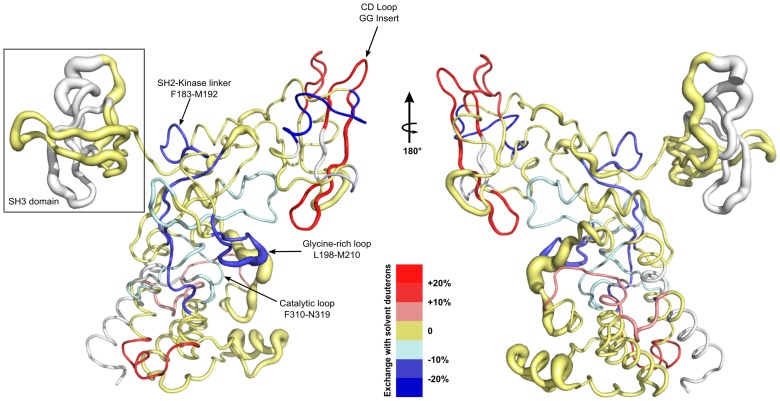
A combined representation of Molecular Dynamics simulations (MD) and Deuterium Exchange-Mass Spectrometry (DXMS) data showing the effects of SH2 domain's CD loop elongation mapped on a worm representation of the crystal structure of full length Csk. DXMS data are shown according to the key at the bottom. Protected regions are represented by the blue gradient for the variant with respect to wild type while deprotected regions are represented by the red gradient. Regions in gold represent probes that show no significant difference in protection between the wild type and variant and gray represents absence of reliable DXMS probes. Protection criteria and classification are determined as described in figures 3 and 4 and the methods section. MD data are represented by the most statistically relevant divergence in root mean square fluctuation (RMSF) for the backbone heavy atoms represented by the thickness of the worm at any given position. Important motifs and domains are annotated based on the crystal structure of Csk.

For an inherently complex system like Csk, where cross-domain communications are essential for function, computationally robust and statistically relevant tools are essential. Thus, to understand the coupling of distal residues to catalytic hubs in Csk, we employed Molecular Dynamics (MD) studies [42]–[45] by running four independent simulations for more than 100 ns each for the two proteins. The eight simulations were first aligned to the crystallographic structure [34] used to build the system (PDB entry 1K9A; chain A). The root mean square fluctuations (RMSF) were computed for each individual simulation and then averaged for all trajectories. In Figure 5, we combine results of MD runs and DXMS experiments into a hybrid representation of the fluctuations observed in the full length protein in this study. The relative difference we report here is the absolute divergence between the averaged magnitudes of fluctuations between the variant and wild type simulations. Thus, the thickness of the tube represents statistically significant divergence in fluctuations (backbone heavy atom RMSF values) observed in the MD simulations for the two proteins whereas the color gradient represents the observed protection differences between wild type and variant Csk in DXMS.

Interestingly, while the SH3 domain shows no significant differences in DXMS, simulations reveal a likely concerted motion of the SH3 domain as a whole unit giving rise to observed fluctuations as most of the residues exhibit high RMSF values and a form of periodicity (Fig. 5 boxed). MD fluctuations within the SH2 domain correlated well with hydrogen deuterium exchange results in several regions. Around the elongated CD loop, DXMS shows faster exchange in the variant relative to the wild type. Inspection of the MD trajectories shows that the reduced Cys122 is stabilized in the variant where this local stabilization is achieved by the formation of two hydrogen bonds: 1) between the backbone carbonyl of the cysteine residue and the backbone N-H group of the inserted Gly125′, and 2) between the backbone N-H group of Cys122 and the carbonyl backbone of Lys125. These bonds formed only transiently during the wild type simulations likely because of the shorter CD turn. While the short loop length in wild type may contribute to strain leading to higher RMSF values in the wild type, the formation of local backbone loop contacts may explain the corresponding loss of protection upon Gly-Gly insertion, which would allow for longer exposure of *surrounding* amide protons to solvent. Stabilizing effects are seen in other regions that correspond to distal motifs and are part of the catalytic machinery. Such long-range coupled effects underlie the intrinsic cross-domain communications that are integral to Csk's function as a multidomain enzyme [15]. Notably, the conserved Arg313 of the catalytic loop (*i*−1 to the catalytic base) is overall more stable in the variant trajectories than in the wild type. This is consistent with an *activation* loop that displays larger ranges of motion in the wild type that may sterically influence the apparent stability of the proximal Arg313 of the catalytic motif. Moreover, Phe183 is an important allosteric modulatory site in Csk [30]. While our simulations do not reveal significant divergence for this residue between the wild type and the variant, DXMS data indicate protection in the region (Figure 4, SH2-Kinase linker). Regions that do not show overlap or significant correlation between MD and DXMS most likely undergo motions on time scales that were not accessible in our 100 ns simulations, or are regions that lacked sufficient reliable probes in our DXMS experiments. Thus, the computational and experimental techniques are complementary in this study. It is worth noting that residues 341–346 of the activation loop were modeled using *Prime* from the Schrodinger suite [46] since they are missing from the starting crystal structure. DXMS studies of wild type Csk indicate high mobility of this loop and MD studies reveal that the identically modeled loop has an overall larger range of motion in the wild type simulations than in the variant, as did a small helix in the kinase domain (residues 393 to 403). Like the SH3 domain, this helix was observed moving as a unit and is proximal to the identified [47] substrate-docking site. On the whole, our experimental and computational studies highlight the importance of distal sites communications and sensitivity of Csk's activity to allosteric motif modifications; they also emphasize the usefulness of utilizing such complementary approaches in detecting intramolecular signaling networks.

Csk's kinase activity is dependent on its kinase domain's interactions with the modular SH2 and SH3 domains. Previous mutagenesis and Hydrogen-Deuterium exchange experiments revealed the presence of communication across the protein framework by identifying important (and often conserved) residues in the linkers and domain interfaces that are akin to signaling hubs [21], [23], [30], [48]. In this study, we utilize experimental and computational techniques to investigate the importance of an allosteric site in Csk's SH2 domain. It's interesting to note that in a recent study of a longer CBP peptide scaffold mimic, the BC loop is shown to be a novel, non-canonical binding motif that is important for tumor suppression [49]. Our MD studies show increased fluctuations in that region upon CD loop elongation highlighting the fact that conventional models do not fully explain SH2 domain regulation of Csk's activity. Indeed our data show that the enzyme's native dynamics are sensitive to changes in a region that is even farther removed from the active site (45 Å) and respond to modifications to a variable segment in homologous SH2 domains. In addition to the SH2-kinase linker, the αC and β3 in the kinase small lobe are thought to be important anchors for residues that are essential for activation of Csk by its SH2 domain [23]. The reduced flexibility in the kinase small lobe is likely to have arisen from changes routed to the active site via these motifs. We propose that the reduced flexibility observed in the small lobe of the kinase domain is responsible for the observed reduction in catalytic activity affecting *k_cat_* but not substrate recognition. A conformational change in Csk was previously proposed to be a rate limiting step in the catalytic cycle [36], the observed changes in native motions are likely involved in that same rate limiting step for the variant. Moreover, the induced dichotomy in dynamics between the SH2 and kinase domains is in agreement with previous observations where mechanical stress in Csk was shown to be balanced along the framework of the protein [26]. Relieving loop strain and introducing new contacts in the SH2 domain could lead to disruption in balanced native stress within the SH2 domain which induces compensatory effects in the highly coupled framework of Csk. Thus, stresses seem to be tuned in Csk where increases in some regions are balanced by decreases in strain at other regions. Studies on dihydrofolate reductase have uncovered similar behavior in that enzyme where a network of coupled motions work cohesively to facilitate substrate binding and catalysis [50].

The scaffolding of Csk-CBP-SFKs system is thought to be essential for SFK inactivation. In modular proteins like Csk and Src, the SH2 domain is an essential molecular component for activation (Csk) or inactivation (SFKs) [51]. While the manipulation of scaffolding signaling pathways by domain swapping was proposed as a way to reshape cellular behavior [52], the approach requires switching whole components to achieve new signaling routes or alternative pathways. Coupling this strategy to conservative motif modifications (functional loop length engineering) that alter intrinsic activity may produce a more efficient signaling modulation and selectivity.

## Materials and Methods

### Protein Expression and Purification

The Csk SH2 domain and its variant (SH2-GG) constructs were expressed in *E. coli* strain BL21(DE3) in LB media. Cells were grown at 30°C, induced with 0.5 mM isopropyl-1-thiol-β-D-galactopyranoside (IPTG), and harvested after 18 hrs of incubation at 25°C. After cell lysis, WT-SH2 cleared lysate was loaded on a charged Ni^2+^ affinity column equilibrated with binding buffer (20 mM Tris, 500 mM NaCl, pH 7.9) and placed on a rocking platform at 4°C for 1 hr. The column was then washed with 5 resin volumes using wash buffer (20 mM Tris, 500 mM NaCl, 40 mM Imidazole, pH 7.9) and 2 resin bed volumes of elution buffer (20 mM Tris, 500 mM NaCl, 400 mM Imidazole, pH 7.9) was used to elute the proteins. The SH2-GG variant was purified under denaturing conditions where the cell pellets were solubilized in Buffer B (QIAGEN The QIAexpressionist, 5th edition) by stirring at room temperature for 45–60 min. The cleared lysate was loaded on a charged Ni^2+^ affinity column equilibrated with Buffer B and rocking at 4°C for 1 hr. Refolding was done by flowing 5 resin bed volumes of native binding buffer over the column. Subsequent purification steps were identical to that of WT-SH2 above.

C-terminus (His)_6_ tagged full length Csk enzymes were expressed in *E. coli* strain BL21(DE3) (Novagen) expression vector were grown at 30°C, induced with 0.5 mM isopropyl-1-thiol-β-D-galactopyranoside, and harvested after 18 hrs of incubation at 25°C. The wild type and variant Csk enzymes were purified by affinity chromatography as follows: Harvested cell pellets was resuspended in binding buffer (50 mM Tris, 20 mM Imidazole, pH 8.0) and lysed by sonication. The cell lysate was then cleared by centrifugation at 20000× *g* for 20 min. The cleared supernatant was applied to a Ni^2+^ affinity column packed with Ni-NTA resin pre-charged with 100 mM NiSO_4_ solution and equilibrated with the binding buffer. The sample was placed on a rocking platform in the cold room for 1 hr. The column was washed with 5 resin volumes of wash buffer (50 mM Tris, 60 mM Imidazole, pH 8.0). 2 resin bed volumes of elution buffer (50 mM Tris, 300 mM Imidazole, pH 8.0) was used to elute the proteins. The purified full-length enzymes were dialyzed against 50 mM Tris (pH 8.0), 150 mM NaCl, 10% (v/v) glycerol, 2 mM DTT and then concentrated and stored at −80°C. Purity was assessed by SDS-PAGE analysis and concentrations determined via UV absorption at 280 nm using an extinction coefficient of 74425 M^−1^ cm^−1^.

Catalytically defective Src (kdSrc) was expressed and purified as described previously [35] with the following changes: 1) A Ni^2+^ affinity column was used in binding the (His)_6_ tagged kdSrc 2) An extensive washing step with the detergent-containing binding buffer was performed to remove all the non-specific binding of the co-expressed chaperon complex.

### Stability Measurements

Equilibrium unfolding titrations were measured by monitoring the average fluorescence wavelength of a single tryptophan in the SH2 domain. Fluorescence emission spectra were collected from 300 to 450 nm using a spectroflourimeter. Samples were prepared at constant protein concentration in a buffered solution (Tris) containing varying concentrations of denaturant ranging from 0 to 5.0 M (Gdn-HCl).

### NMR Experiments

NMR samples were prepared by growing in modified M9 medium containing [^15^N] Ammonium Sulfate (2 g/L) and/or [^13^C] glucose (2 g/L) for uniformly ^15^N-labeled and ^13^C,^15^N-labeled protein, respectively. NMR experiments were run on a DMX 500 spectrometer (Varian) using standard pulse sequences in the Varian BioPack. Assignments of the SH2 domains were done using standard assignment methods [53]. NMR data were processed using the NMRPipe package [54] and analyzed using Sparky [55] and CARA software [56].

The purified Csk SH2 constructs were dialyzed against 10 mM phosphate buffer (pH 7.0), 50 mM NaCl, ±2 mM DTT then concentrated to approximately 0.7 mM for NMR experiments. All non-deuterated protein samples contained 10% (v/v) D_2_O for NMR experiments. Purity was assessed by SDS-PAGE analysis and concentrations determined via UV absorption at 280 nm using an extinction coefficient of 16055 M^−1^ cm^−1^ (ExPasy-Protparam). NMR-detected Hydrogen-Deuterium exchange in SH2 constructs was monitored by measuring intensities of assigned ^1^H-^15^N cross peaks by recording a series of ^1^H-^15^N HSQC NMR spectra (50 total, 21 min each) immediately after protein deuteration. Typically, a 0.8 mM protein sample (in 10 mM Na_2_HPO_4_, 50 mM NaCl, ±2 mM DTT, pH 7.5) is split into a non-deuterated (ND) and fully-deuterated (FD) samples. The total volume in ND samples is brought up to 0.6 mL with buffer and 10% (v/v) D_2_O for spectrometer referencing. The FD samples are prepared by applying 300 µL of the protein sample to a Quick Spin Protein Column (Roche) pre-equilibrated with the deuterated (99.9% D_2_O) buffer (10 mM Na_2_HPO_4_, 50 mM NaCl, ±2 mM d_10_-DTT, pD 7.9) then centrifuging the column for 3 min at 750× *g*. The collected deuterated eluate is brought up to volume by adding deuterated buffer and 10% (v/v) D_2_O to maintain buffer composition as the ND samples.

### Kinase Activity Assay for Src Phosphorylation by Csk and Its Variants

Time-dependent phosphorylation of kinase dead Src (kdSrc) by wild type or variant Csk enzymes was typically carried out in assay buffer: 100 mM MOPS (pH 7.0), 100 mM KCl, 10 mM MgCl_2_, 100 µM [γ-^32^P]ATP (4000–6000 cpm pmol^−1^) and 10 mM DTT at lab temperature (23°C). Reactions were initiated by adding ATP to the master mix containing all enzymes and reagents. 10 µL from the master mix reactions was quenched directly into 10 µL of 2× SDS loading buffer at several time points. The quenched samples were analyzed by SDS-PAGE and bands corresponding to the phosphorylated kdSrc were excised from the dried gels and quantified on the ^32^P channel in liquid scintillator. The specific activity of [γ-^32^P]ATP was determined by measuring the total counts of the reaction mixture. The time-dependent concentration of ^32^P-Src was then determined by considering the total counts per minute (CPM), the specific activity of the reaction mixture, and the background phosphorylation. Initial velocity reactions were typically performed in 10 µL volumes at 23°C and initiated by adding Csk enzymes to substrates and allowing the reaction to proceed for 6 minutes before quenching with 2× SDS loading buffer. The quenched samples were analyzed as described above.

### Deuterium Exchange-Mass Spectrometry (DXMS)

#### DXMS operation

The instrument setup and operation was previously described [38]. All frozen samples were thawed and run using the conditions determined during fragmentation optimization.

#### Fragmentation conditions

The initial conditions for the sample composition and instrument parameters were determined before starting the exchange time course experiments. 1.4 mg/ml stocks of wild type or variant Csk were diluted with storage buffer (50 mM Tris, 150 mM NaCl, 10% glycerol, 2 mM DTT, pH 7.0) at room temperature and quenched with 0.5% formic acid, 16.6% glycerol, and 3.2 M Guanidine-HCl (quench buffer) at 0°C then immediately frozen on dry ice and stored at −80°C until analysis.

#### Deuterium on-exchange experiments

The exchange time course experiments for wild type and variant Csk were all performed simultaneously at lab temperature (23°C) with the following procedure. A full time course experiment was initiated by adding 77 µL of protein in H_2_O buffer (50 mM Tris, 150 mM NaCl, 10% glycerol, 2 mM DTT, pH 7.0) to 187 µL of the equivalent deuterated exchange buffer for a final D_2_O of 71%. The deuterated exchange buffer (50 mM d-Tris, 150 mM NaCl, 10% glycerol, 2 mM deuterated DTT) was prepared using 99.9% D_2_O and adjusted to pD 7.4 with DCl. The exchange was monitored over the course of 24 hours at intervals of 0.1, 0.5, 1, 5, 15, 60, 120 and 1440 min. Aliquots of 24 µL from the master reaction were removed and quenched in pre-chilled high pressure liquid chromatography vials containing 6 µL of quench buffer. The vials were sealed and frozen over dry ice, then stored at −80°C. The in-exchange control consisted of the protein added directly to the pre-chilled deuterated and quench buffers, then immediately followed by the normal sample preparation procedure. The back exchange control was determined by incubating the samples in 0.5% formic acid in D_2_O for 24 hours All samples were injected and run on the instrument with the same conditions listed in the fragmentation optimization screening. Data for the time course exchanges were acquired in the MS1 mode.

#### Sequence identification of peptide fragments

The most likely identity of the parent peptide ions was determined using the SEQUEST software program (Thermo Finnigan, Inc.) and MS1 and MS2 data. The quality of each peptide was monitored by individually examining each measured isotopic envelope spectrum for the entire time course exchange. The deuterium content was calculated for each time point by using specialized software as previously described [57].

### Computational Methods

The structure was selected from the first active conformation (chain A) of the crystal refined structure with PDB ID 1K9A [34]. Missing loops were modeled with the PRIME module in the Schrodinger suite and residues were changed to match the human CSK sequence [46]. The H++ web-server (http://biophysics.cs.vt.edu/H) was used to predict the protonation state of the residues in the structure at a pH of 7.0. The CSK system was solvated in a cubic box and additional Na^+^ and Cl^−^ ions were added to achieve 150 mM physiological conditions. The variant structure was generated by inserting two glycine residues between Gly124 and Lys125. The reduced conditions were modeled by defining residues 122 and 164 as Cys residues. The water was modeled with the 4-particle TIP4P-Ew force field [58], which was previously shown to better describe the rotational motion of proteins [59] than the related 3-particle water model, TIP3P [60]. SHAKE was applied to all bonds involving hydrogen atoms [61]. Minimization was applied to the resulting structure to remove any clashes. Harmonic positional restraints of strength 10 kcal/mol/Å^2^ were applied to the protein backbone atoms, keeping the pressure at 1 atm and increasing the temperature from 10 K to 300 K as a linear function of time over the course of 1.2 ns, using Berendsen temperature and pressure control algorithms with relaxation times of 0.5 picoseconds for both the barostat and the thermostat [62]. Restraints were removed and a 6 ns simulation was performed at constant isotropic pressure of 1 atm and a temperature of 300 K. We used a 10 Å cutoff radius for range-limited interactions, with Particle Mesh Ewald electrostatics for long-range interactions [63].

The production simulations of CSK were carried out using NVT conditions. A Langevin thermostat was used to maintain the temperature at 300 K with a collision frequency of 2 ps. The simulation time step was 2 fs and snap shots were saved every 2 ps. For all simulations performed the first 5 ns of the NVT production runs were excluded allowing the systems to fully equilibrate for further analysis. All simulations were performed using the PMEMD module within the Amber11 simulation package. Production NVT runs were performed on GTX580 GPUs using the Amber11 pmemd.CUDA engine [44]–[45]. Four independent 100 ns simulations were run for each the wild type and the Gly-Gly insert Csk variant.

## Supporting Information

Figure S1 **Equilibrium Unfolding Titration of wild type and variant Csk SH2 domains.** The stability of the SH2 domains is measured as a function of chemical denaturant concentration. Csk's variant SH2 domain (square) is slightly less stable than the wild type (circle) by a ΔΔG of ∼1.5 kcal/mol. The calculated value was determined as described in methods.
(TIF)

Figure S2 **Comparison of the global fold of SH2 domains.** An overlayed view of ^1^H-^15^N Heteronuclear Single Quantum Correlation (HSQC) spectra of wild type (pink) and variant (blue) SH2 domains. The observed chemical shift dispersion indicates retention in globular domain fold. Differences in backbone amide resonances indicate that regions around the insertion site are affected by the CD loop elongation. For clarity, backbone amide assignments are shown for the variant (SH2-GG) domain only.
(TIF)

Figure S3 **NMR detected Hydrogen-Deuterium exchange in SH2 and domain dynamics.** Residue-specific Hydrogen-Deuterium exchange (HDx) effects mapped on the structure of Csk's SH2 domain. Red indicates faster exchange with solvent deuterons in the variant domain with respect to wild type while blue indicates slower exchange. Yellow indicates backbone amides that exhibit the same degree of protection while residues in gray indicate absence of probes whose exchange is too fast to measure. The unique disulfide bridge in Csk's SH2 domain and the CD loop are shown.
(TIF)

Figure S4 **Circular Dichroism spectra show the full-length variant is folded.** Wild type Csk (solid) and the variant (dashed) have similar CD signature spectra. The two minima at equal molar ellipticity value is indicative of a folded enzyme with dominant alpha-helical secondary structure.
(TIF)

Figure S5 **Reduced kinase activity of full length Csk towards a generic substrate.** Wild type Csk's (circle) and the variant's (square) kinase activity was monitored in a [γ-^32^P]ATP coupled radioactive assay in which a generic kinase substrate (polyEY) is phosphorylated as a function of time. The reactions typically included 200 nM Csk, 2 mg/mL polyEY, and 50 µM ATP at 23°C.
(TIF)

Figure S6 **Km(s) of substrates.** Rate of substrate phosphorylation is measured as a function of substrate concentration for Csk's physiological substrate Src (top) and ATP (bottom). Reaction rates were determined after incubating Csk or the variant (100 nM) with kdSrc (20 µM) for 6 minutes.
(TIF)

Figure S7 **Activation of Csk by CBP phosphopeptide.** Percent activation of wild type Csk (circle) and the variant (square) by CBP phosphopeptide was monitored using kdSrc as a substrate. Equal amounts of wild type Csk and the variant (60 nM) were mixed with 100 µM [γ-^32^P]ATP, 20 µM kdSrc, and various amounts of CBP phosphopeptide (0–10 µM) at 23°C. The activities were measured as a function of phosphopeptide concentration and the rates were normalized to the zero point values for each enzyme (100%).
(TIF)

Figure S8 **Representative time-dependent solvent deuterium incorporation.** Mass envelope shift of a representative peptide probe in wild type (WT-CSK, bottom) and variant (CSK-GG, top) is indicative of the differences observed for time-dependent incorporation of solvent deuterons into the probe E93-F104. The key on the right shows color-coded time-points corresponding to mass spectra in each graph that show deuteration mass envelope of the same peptide in both the wild type and the variant Csk.
(TIF)
