# Supplementary figures and images for: Distal Loop Flexibility of a Regulatory Domain Modulates Dynamics and Activity of C-Terminal Src Kinase (Csk)
Source: PLoS Comput Biol. 2013 Sep 5;9(9):e1003188. doi: 10.1371/journal.pcbi.1003188 (PMC3764022; doi:10.1371/journal.pcbi.1003188)

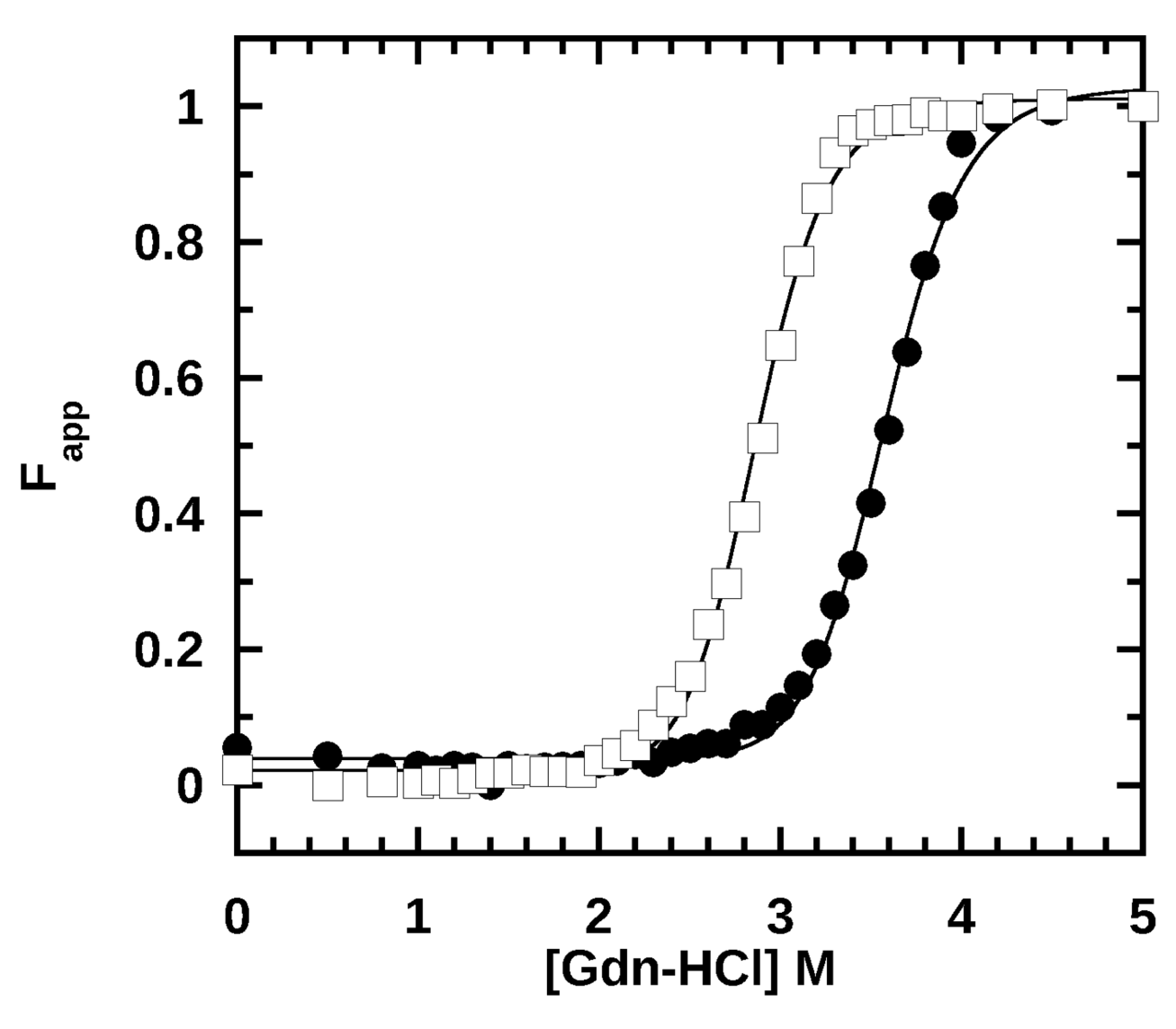

Supplement: Figure S1 — Equilibrium Unfolding Titration of wild type and variant Csk SH2 domains. The stability of the SH2 domains is measured as a function of chemical denaturant concentration. Csk's variant SH2 domain (square) is slightly less stable than the wild type (circle) by a ΔΔG of ∼1.5 kcal/mol. The calculated value was determined as described in methods. (TIF) [file pcbi.1003188.s001.tif]

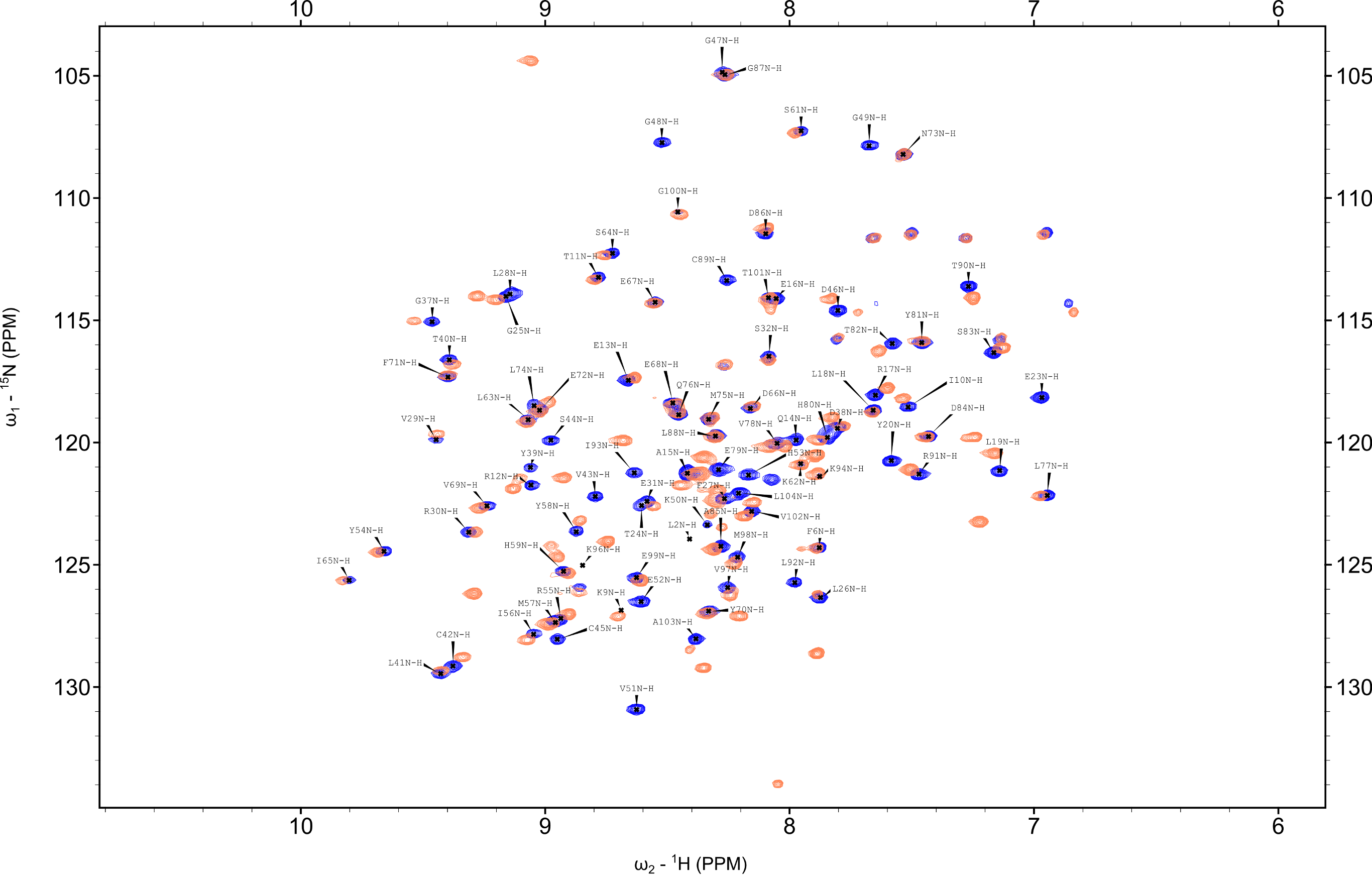

Supplement: Figure S2 — Comparison of the global fold of SH2 domains. An overlayed view of 1H-15N Heteronuclear Single Quantum Correlation (HSQC) spectra of wild type (pink) and variant (blue) SH2 domains. The observed chemical shift dispersion indicates retention in globular domain fold. Differences in backbone amide resonances indicate that regions around the insertion site are affected by the CD loop elongation. For clarity, backbone amide assignments are shown for the variant (SH2-GG) domain only. (TIF) [file pcbi.1003188.s002.tif]

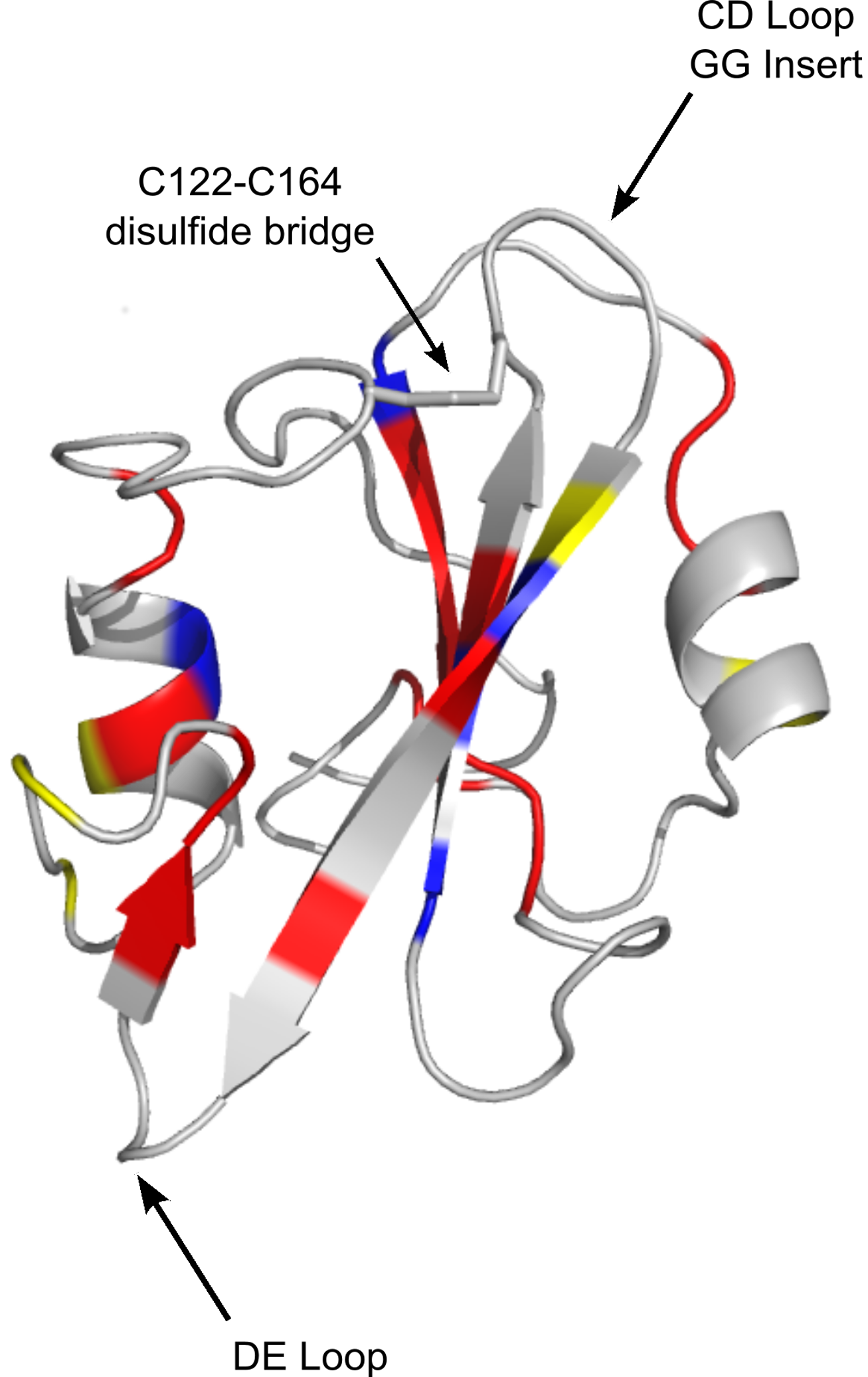

Supplement: Figure S3 — NMR detected Hydrogen-Deuterium exchange in SH2 and domain dynamics. Residue-specific Hydrogen-Deuterium exchange (HDx) effects mapped on the structure of Csk's SH2 domain. Red indicates faster exchange with solvent deuterons in the variant domain with respect to wild type while blue indicates slower exchange. Yellow indicates backbone amides that exhibit the same degree of protection while residues in gray indicate absence of probes whose exchange is too fast to measure. The unique disulfide bridge in Csk's SH2 domain and the CD loop are shown. (TIF) [file pcbi.1003188.s003.tif]

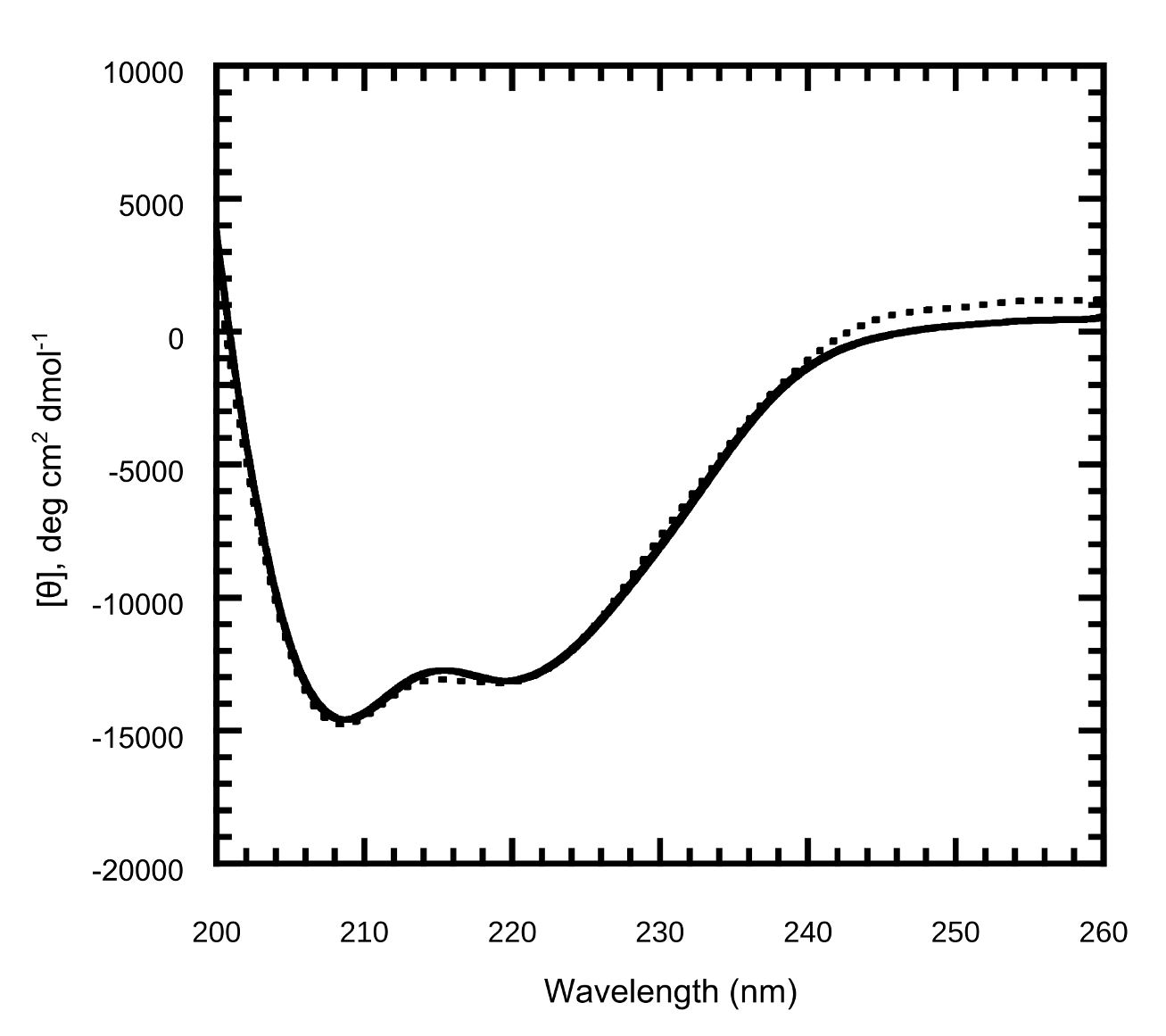

Supplement: Figure S4 — Circular Dichroism spectra show the full-length variant is folded. Wild type Csk (solid) and the variant (dashed) have similar CD signature spectra. The two minima at equal molar ellipticity value is indicative of a folded enzyme with dominant alpha-helical secondary structure. (TIF) [file pcbi.1003188.s004.tif]

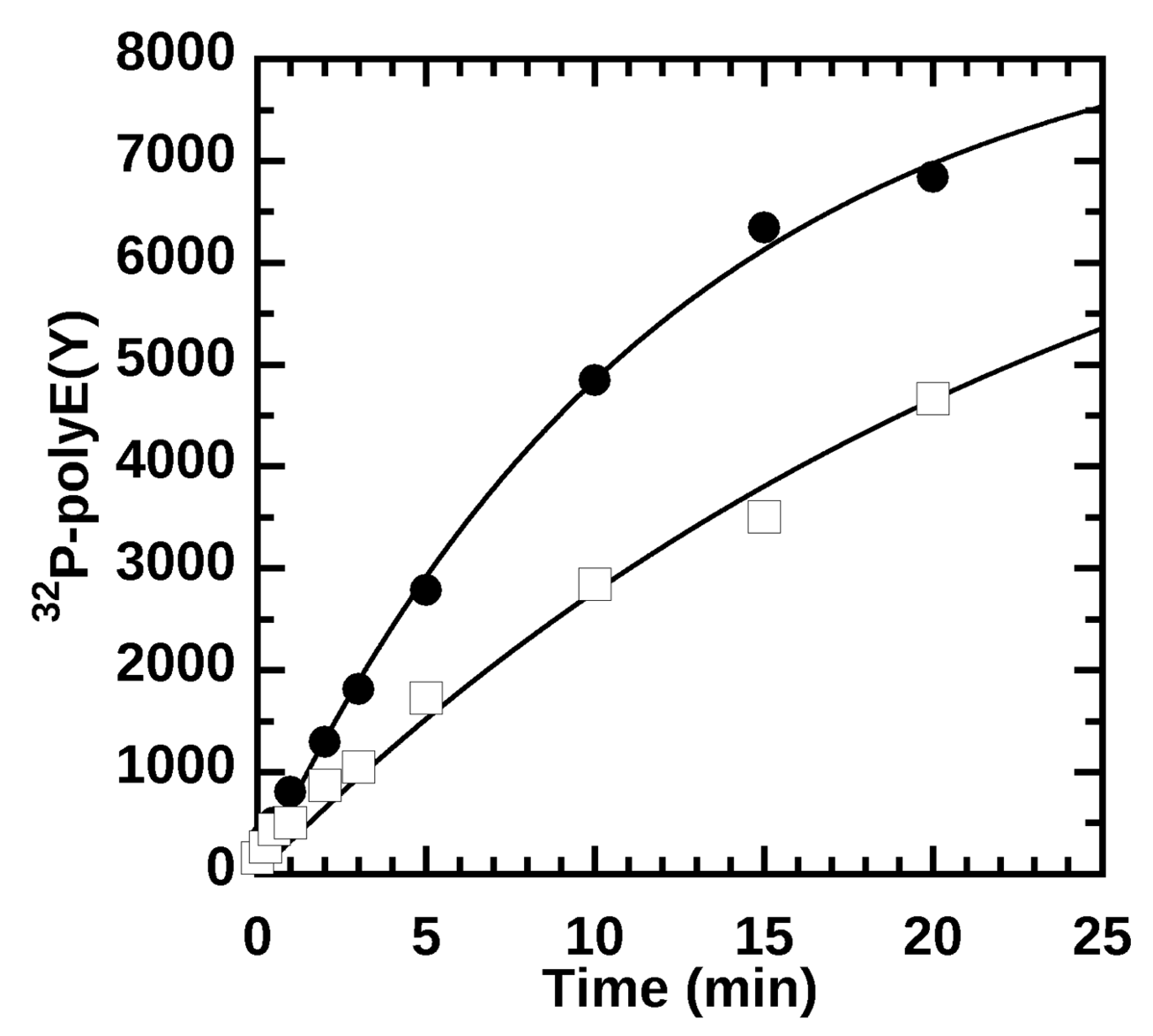

Supplement: Figure S5 — Reduced kinase activity of full length Csk towards a generic substrate. Wild type Csk's (circle) and the variant's (square) kinase activity was monitored in a [γ-32P]ATP coupled radioactive assay in which a generic kinase substrate (polyEY) is phosphorylated as a function of time. The reactions typically included 200 nM Csk, 2 mg/mL polyEY, and 50 µM ATP at 23°C. (TIF) [file pcbi.1003188.s005.tif]

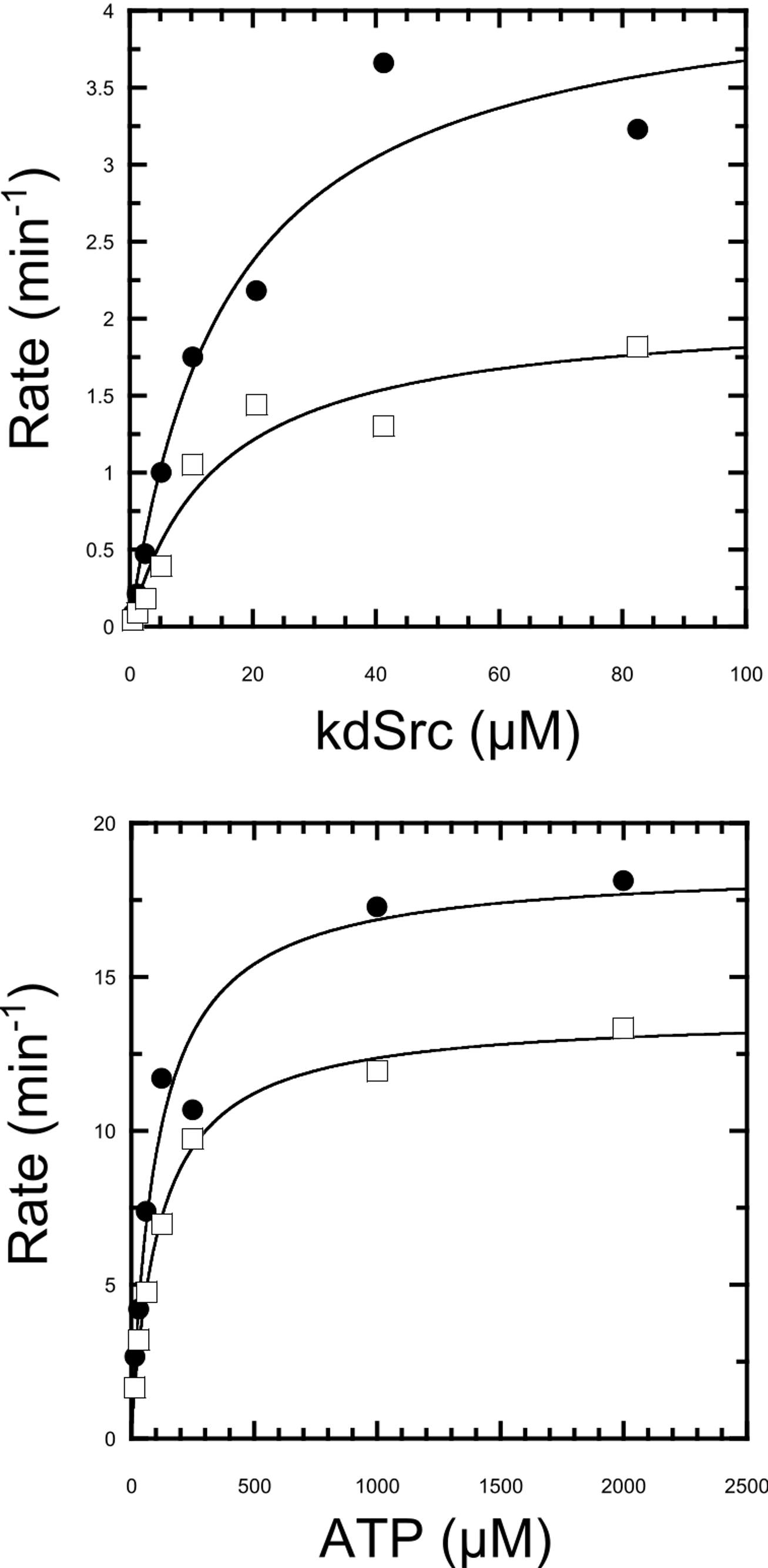

Supplement: Figure S6 — Km(s) of substrates. Rate of substrate phosphorylation is measured as a function of substrate concentration for Csk's physiological substrate Src (top) and ATP (bottom). Reaction rates were determined after incubating Csk or the variant (100 nM) with kdSrc (20 µM) for 6 minutes. (TIF) [file pcbi.1003188.s006.tif]

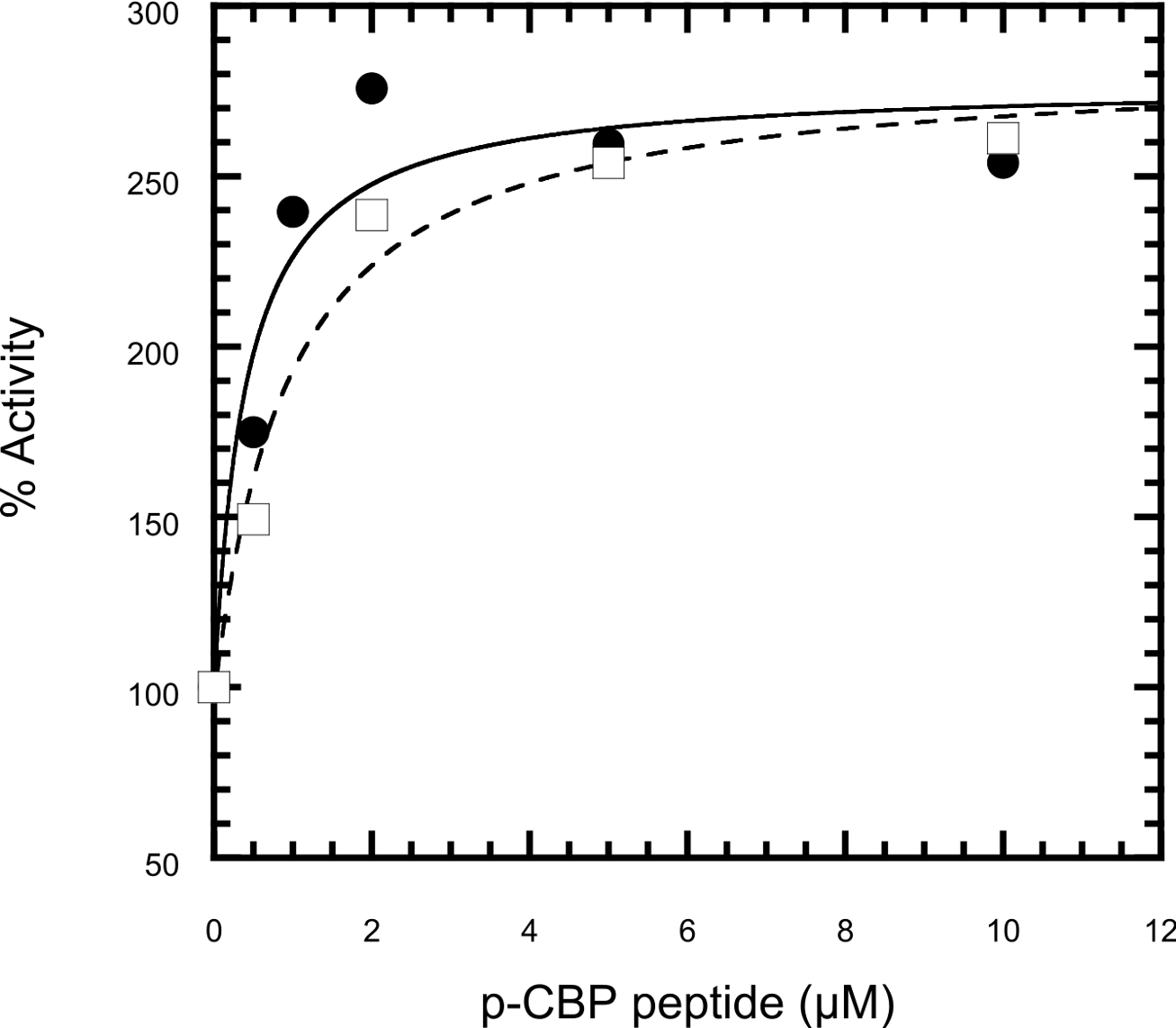

Supplement: Figure S7 — Activation of Csk by CBP phosphopeptide. Percent activation of wild type Csk (circle) and the variant (square) by CBP phosphopeptide was monitored using kdSrc as a substrate. Equal amounts of wild type Csk and the variant (60 nM) were mixed with 100 µM [γ-32P]ATP, 20 µM kdSrc, and various amounts of CBP phosphopeptide (0–10 µM) at 23°C. The activities were measured as a function of phosphopeptide concentration and the rates were normalized to the zero point values for each enzyme (100%). (TIF) [file pcbi.1003188.s007.tif]

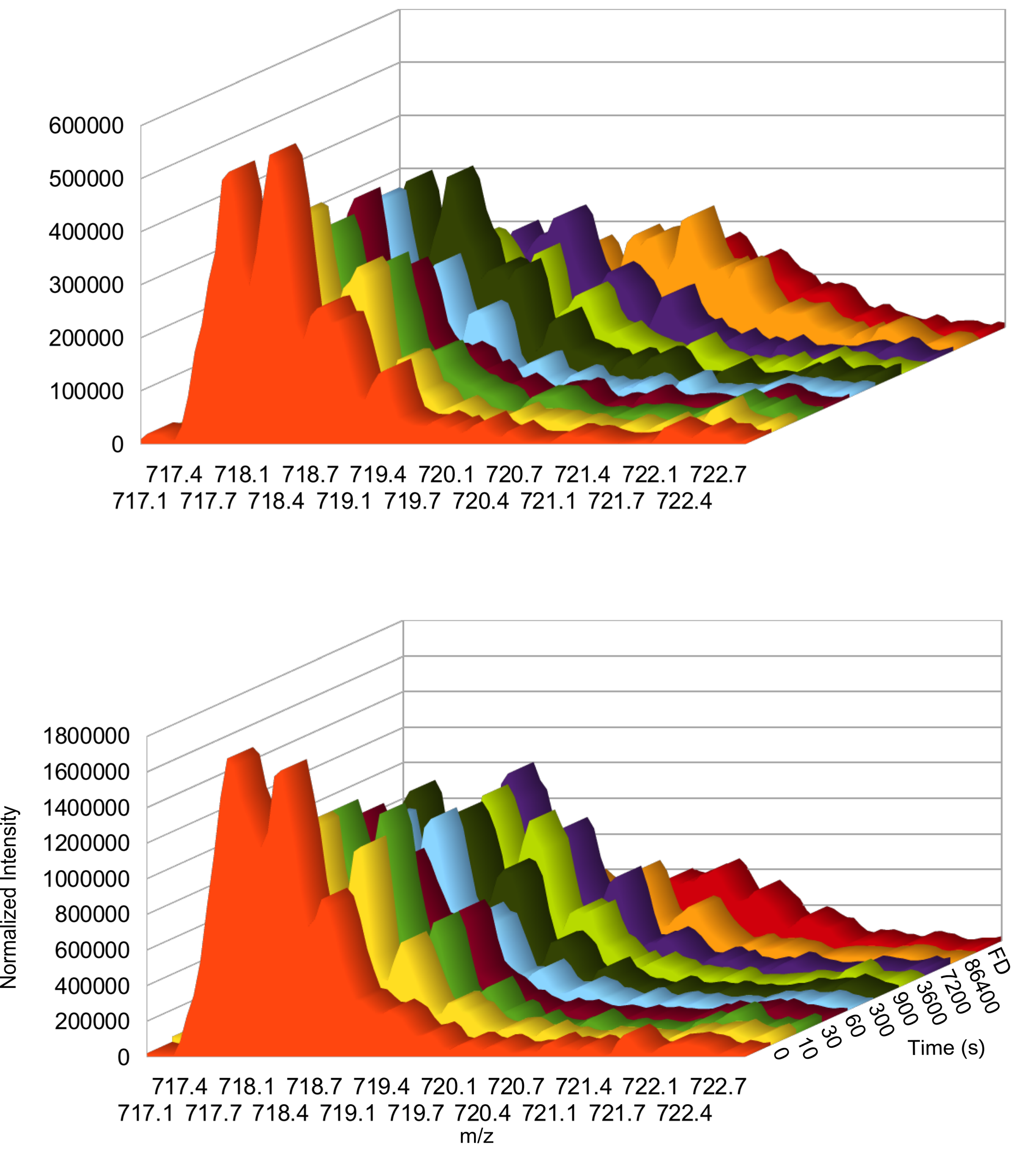

Supplement: Figure S8 — Representative time-dependent solvent deuterium incorporation. Mass envelope shift of a representative peptide probe in wild type (WT-CSK, bottom) and variant (CSK-GG, top) is indicative of the differences observed for time-dependent incorporation of solvent deuterons into the probe E93-F104. The key on the right shows color-coded time-points corresponding to mass spectra in each graph that show deuteration mass envelope of the same peptide in both the wild type and the variant Csk. (TIF) [file pcbi.1003188.s008.tif]
